# Supplementary material for: Carriage of ESBL-producing Enterobacterales in wastewater treatment plant workers and surrounding residents — the AWARE Study
Source: Eur J Clin Microbiol Infect Dis. 2021 Dec 13:1–16. Online ahead of print. doi: 10.1007/s10096-021-04387-z (PMC8667530; doi:10.1007/s10096-021-04387-z)
Supplement: Supplementary file 1 — Supplementary file1 (PDF 482 KB) [file 10096_2021_4387_MOESM1_ESM.pdf]

# Supplementary Information

## Carriage of ESBL-producing Enterobacterales in wastewater treatment plant workers and surrounding residents - The AWARE Study

Daloha Rodríguez-Molina[1,2,3], Fanny Berglund[4,5], Hetty Blaak[6], Carl-Fredrik Flach[4,5], Merel Kemper[6], Luminita Marutescu[7,8], Gratiela Pircalabioru Gradisteanu[7,8], Marcela Popa[7,8], Beate Spießberger[9,10,11], Tobias Weinmann[1], Laura Wengenroth[1], Mariana Carmen Chifiriuc[7,8], D. G. Joakim Larsson[4,5], Dennis Nowak[1,12], Katja Radon[1], Ana Maria de Roda Husman[6], Andreas Wieser[9,10,11], Heike Schmitt[6].

1: Institute and Clinic for Occupational, Social and Environmental Medicine; University Hospital, LMU Munich, Munich, Germany.

2: Institute for Medical Information Processing, Biometry, and Epidemiology – IBE, LMU Munich, Munich, Germany.

3: Pettenkofer School of Public Health, Munich, Germany.

4: Department of Infectious Diseases, Institute of Biomedicine, The Sahlgrenska Academy, University of Gothenburg, Gothenburg, Sweden.

5: Centre for Antibiotic Resistance Research (CARe), University of Gothenburg, Gothenburg, Sweden.

6: Centre of Infectious Disease Control, National Institute for Public Health and the Environment, Bilthoven, The Netherlands.

7: Department of Microbiology and Immunology, Faculty of Biology, University of Bucharest and the Academy of Romanian Scientists, Bucharest, Romania.

8: Earth, Environmental and Life Sciences Section, Research Institute of the University of Bucharest, University of Bucharest, Bucharest, Romania.

9: German Centre for Infection Research (DZIF) Partner Site Munich.

10: Max von Pettenkofer Institute, Faculty of Medicine, LMU Munich.

11: Department of Infectious Diseases and Tropical Medicine, LMU University Hospital Munich, Munich, Germany.

12: Comprehensive Pneumology Center Munich (CPC-M), German Center for Lung Research (DZL), Munich, Germany.

**Corresponding author**: Daloha Rodríguez-Molina; Occupational and Environmental Epidemiology and & NetTeaching Unit; Institute and Clinic for Occupational, Social and Environmental Medicine;

University Hospital, LMU Munich; Ziemssenstr. 5, D-80336 Munich, Germany; Tel.: +49 (89) 4400-52358 Fax.: +49 (89) 4400-54954; e-mail: daloha.rodriguez_molina@med.uni-muenchen.de

# Contents


- Online Resource Table 1: Descriptive characteristics of ESBL-producing *E. coli* carriers by country, n = 1940, AWARE Study, 2021
- Online Resource Table 2: Comparison of models estimating the effect of participation group (wastewater treatment plant -WWTP- worker, nearby resident, distant resident) as a proxy for exposure routes (ingestion of droplets, hand-to-mouth contact, or inhalation of aerosols) in and around the local WWTP on the presence of ESBL-producing E. coli in stool samples, AWARE Study, 2021
- Online Resource Table 3: Multiple imputation diagnostics - Traditional (unweighted) logistic regression models, complete cases vs. imputed, AWARE Study, 2021
- Online Resource Table 4: Interaction model with an interaction term for participation group with country for the carriage of ESBL-producing E. coli in comparison to crude estimates from a traditional unweighted logistic regression model, n = 1940, AWARE Study, 2021
- Online Resource Fig. 1: Comparison of models estimating the effect of participation group (wastewater treatment plant -WWTP- worker, nearby resident, distant resident) as a proxy for exposure routes (ingestion of droplets, hand-to-mouth contact, or inhalation of aerosols) in and around the local WWTP on the presence of ESBL-producing E. coli in stool samples (all estimates are shown), AWARE Study, 2021. Models adjusted for age, sex, education, country, travels to high risk areas, working with human tissues, antibiotic use, farm visits, hospital visits as patient and hospital visits as a professional. IPW: Inverse Probability Weighted model. ref. = Reference level. Travel to high risk areas for AR in the past year includes travels to North Africa, Sub-Saharan Africa, Asia, Central and South America, as well as the European countries Italy, Greece, Bulgaria and Slovenia. Crude: Model with only the given variable, ignoring potential covariates. Adjusted: Model with the given variable, including all potential covariates in the exposure-outcome relation. Unweighted: Model without applying inverse probability weights (IPW). Weighted: Model applying inverse probability weights (IPW). See text for details
- Online Resource Fig. 2: Comparison of models estimating the effect of participation group (wastewater treatment plant -WWTP- worker, nearby resident, distant resident) as a proxy for exposure routes (ingestion of droplets, hand-to-mouth contact, or inhalation of aerosols) in and around the local WWTP on the presence of ESBL-producing E. coli in stool samples, stratified by participation group, AWARE Study, 2021. Models adjusted for age, sex, education, country, travels to high risk areas, working with human tissues, antibiotic use, farm visits, hospital visits as patient and hospital visits as a professional. IPW: Inverse Probability Weighted model. ref. = Reference level. Travel to high risk areas for AR in the past year includes travels to North Africa, Sub-Saharan Africa, Asia, Central and South America, as well as the European countries Italy, Greece, Bulgaria and Slovenia. Crude: Model with only the given variable, ignoring potential covariates. Adjusted: Model with the given variable, including all potential covariates in the exposure-outcome relation. Unweighted: Model without applying inverse probability weights (IPW). Weighted: Model applying inverse probability weights (IPW). See text for details

*Online Resource Table 1: Descriptive characteristics of ESBL-producing E. coli carriers by country, n = 1940, AWARE Study, 2021*

| | | | Overall | | Germany | | The Netherlands | | Romania | |
|---|---|---|---|---|---|---|---|---|---|---|
| **Variable** | **Missings** | **level** | **Positive** | **p** | **Positive** | **p** | **Positive** | **p** | **Positive** | **p** |
| n | | | 236 | | 26 | | 47 | | 163 | |
| Age, years (median [IQR]) | 0 | | 43 [31, 55] | <0.001 | 38 [31, 51] | 0.051 | 55 [48, 60] | 0.977 | 40 [29, 52] | 0.005 |
| Sex, n (%) | 4 | Female | 122 (13) | 0.959 | 15 (8) | 0.905 | 25 (6) | 0.878 | 82 (28) | 0.919 |
| | | Male | 114 (13) | | 11 (7) | | 22 (5) | | 81 (27) | |
| Highest educational level obtained, n (%)[a] | 8 | Low | 75 (11) | 0.068 | 6 (4) | 0.189 | 15 (4) | 0.033 | 54 (41) | <0.001 |
| | | High | 161 (14) | | 20 (9) | | 32 (8) | | 109 (24) | |
| Participation group, n (%) | 0 | Distant resident[b] | 79 (7) | <0.001 | 21 (8) | 0.259 | 40 (4) | 0.569 | 18 (12) | <0.001 |
| | | Nearby resident[c] | 123 (29) | | 5 (6) | | --- (---)[d] | | 118 (36) | |
| | | WWTP worker | 34 (11) | | 0 (0) | | 7 (6) | | 27 (23) | |
| Work with patients or human tissues in the past year, n (%)[e] | 43 | No | 186 (16) | <0.001 | 15 (7) | 1.000 | 32 (7) | 0.333 | 139 (29) | 0.042 |
| | | Yes | 43 (8) | | 9 (7) | | 15 (5) | | 19 (19) | |
| Hospital visits as a patient in the past year, n (%) | 2 | No | 215 (13) | 0.889 | 22 (7) | 1.000 | 46 (6) | 0.505 | 147 (27) | 0.634 |
| | | Yes | 21 (14) | | 4 (7) | | 1 (2) | | 16 (30) | |
| Hospital visits as a professional in the past year, n (%) | 2 | No | 232 (13) | 0.395 | 25 (7) | 1.000 | 46 (6) | 0.565 | 161 (28) | 0.734 |
| | | Yes | 4 (8) | | 1 (4) | | 1 (7) | | 2 (20) | |
| Use of antibiotics in the past year, n (%) | 4 | No | 177 (13) | 0.703 | 17 (7) | 0.810 | 40 (6) | 0.739 | 120 (27) | 1.000 |
| | | Yes | 56 (14) | | 9 (8) | | 7 (5) | | 40 (27) | |
| Farm visits in the past year, n (%)[f] | 9 | No | 226 (14) | 0.005 | 22 (7) | 1.000 | 42 (6) | 0.799 | 162 (28) | 0.015 |
| | | Yes | 9 (6) | | 4 (6) | | 5 (6) | | 0 (0) | |
| Travel to high risk areas for AR in the past year, n (%) | 18 | No | 169 (14) | 0.031 | 6 (3) | 0.019 | 22 (4) | 0.023 | 141 (30) | 0.010 |
| | | Yes | 62 (11) | | 19 (10) | | 24 (8) | | 19 (17) | |

Notes:
[a]Educational level according to the International Standard Classification of Education (ISCED): Low = ISCED 0-2 (Pre-primary education to Lower secondary education), High = ISCED ≥3 (Upper secondary education to Doctoral or equivalent).
[b]Distant residents live at least 1000 m away from a WWTP
[c]Nearby residents live within a 300 m radius from a WWTP.
[d]No data from nearby residents were collected in the Netherlands.
[e]Work with patients or human tissues in the past year: Includes self-reported contact with patients at work and with human tissues (e.g. blood, urine, sputum, feces, vomit, saliva, or primary cell lines).
[f]Travel to high risk areas for AR in the past year: Includes travels to North Africa, Sub-Saharan Africa, Asia, Central and South America, as well as the European countries Italy, Greece, Bulgaria and Slovenia.
ESBL: Extended-Spectrum Beta-Lactamases; AR: Antibiotic Resistance.

*Online Resource Table 2: Comparison of models estimating the effect of participation group (wastewater treatment plant -WWTP- worker, nearby resident, distant resident) as a proxy for exposure routes (ingestion of droplets, hand-to-mouth contact, or inhalation of aerosols) in and around the local WWTP on the presence of ESBL-producing E. coli in stool samples, AWARE Study, 2021*

| | **Unweighted cOR (95% CI)[a]** | **Unweighted aOR (95% CI)[b]** | **IPW cOR (95% CI)[a,c]** | **IPW aOR (95% CI)[b,c]** |
|---|---|---|---|---|
| Group: Nearby resident [d] | 4.95 (3.63-6.73) | 2.24 (1.5-3.37) | 2.46 (1.65-3.69) | 1.47 (0.83-2.59) |
| Group: WWTP worker | 1.71 (1.12-2.61) | 1.17 (0.74-1.86) | 1.28 (0.82-2) | 0.76 (0.44-1.29) |
| Country: Netherlands | 0.78 (0.47-1.3) | 1.05 (0.61-1.8) | 0.81 (0.47-1.37) | 1.03 (0.58-1.84) |
| Country: Romania | 4.85 (3.04-7.74) | 3.5 (2.13-5.77) | 4.85 (3.02-7.8) | 5.59 (3.29-9.49) |
| Educational level: High[e] | 1.34 (1-1.8) | 1.02 (0.73-1.42) | 1.63 (1.09-2.46) | 1.3 (0.83-2.03) |
| Sex: Male | 1 (0.76-1.32) | 0.99 (0.73-1.35) | 1.07 (0.73-1.57) | 0.98 (0.65-1.48) |
| Age | 0.98 (0.97-0.99) | 0.98 (0.97-1) | 0.99 (0.97-1) | 0.99 (0.98-1.01) |
| Travels to high-risk areas: Yes[f] | 0.73 (0.54-0.99) | 1.27 (0.9-1.8) | 1.65 (1.12-2.43) | 2.06 (1.33-3.19) |
| Work with patients or human tissues: Yes[g] | 0.46 (0.32-0.66) | 0.66 (0.44-0.99) | 0.69 (0.44-1.09) | 0.76 (0.46-1.28) |
| Hospital visits as a patient: Yes | 1.04 (0.65-1.66) | 0.98 (0.58-1.65) | 0.81 (0.42-1.57) | 0.8 (0.4-1.62) |
| Hospital visits as a professional: Yes | 0.57 (0.21-1.59) | 0.83 (0.28-2.48) | 0.72 (0.2-2.62) | 0.76 (0.17-3.36) |
| Use of antibiotics: Yes | 1.07 (0.77-1.48) | 1.04 (0.72-1.5) | 0.97 (0.62-1.54) | 0.93 (0.56-1.52) |
| Farm visits: Yes | 0.4 (0.2-0.77) | 0.7 (0.35-1.39) | 0.97 (0.49-1.92) | 1.23 (0.6-2.51) |

Notes:
[a]cOR: crude odds ratio.
[b]aOR: adjusted odds ratio.
[,c]IPW: Inverse Probability Weighted model.
[d]Nearby residents live within a 300 m radius from a WWTP.
[e]Educational level according to the International Standard Classification of Education (ISCED): Low = ISCED 0-2 (Pre-primary education to Lower secondary education), High = ISCED ≥3 (Upper secondary education to Doctoral or equivalent).
[f]Travel to high risk areas for AR in the past year: Includes travels to North Africa, Sub-Saharan Africa, Asia, Central and South America, as well as the European countries Italy, Greece, Bulgaria and Slovenia.
[g]Work with patients or human tissues in the past year: Includes self-reported contact with patients at work and with human tissues (e.g. blood, urine, sputum, feces, vomit, saliva, or primary cell lines).
ESBL: Extended-Spectrum Beta-Lactamases.
AR: Antibiotic Resistance.

*Online Resource Table 3: Multiple imputation diagnostics - Traditional (unweighted) logistic regression models, complete cases vs. imputed, AWARE Study, 2021*

| | | **Crude models, OR (95% CI)** | | **Adjusted models, OR (95% CI)** | |
|---|---|---|---|---|---|
| | **Missings** | **Complete cases** | **Imputed, n = 1940** | **Complete cases, n = 1707** | **Imputed, n = 1940** |
| Group: Nearby resident [d] | 163 | 5.16 (3.79-7.06) | 4.95 (3.63-6.73) | 2.86 (1.87-4.45) | 2.24 (1.5-3.37) |
| Group: WWTP worker | 163 | 1.56 (1.01-2.37) | 1.71 (1.12-2.61) | 1.09 (0.64-1.83) | 1.17 (0.74-1.86) |
| Country: Netherlands | 163 | 0.79 (0.49-1.32) | 0.78 (0.47-1.3) | 1.37 (0.79-2.45) | 1.05 (0.61-1.8) |
| Country: Romania | 163 | 5 (3.28-7.91) | 4.85 (3.04-7.74) | 3.57 (2.16-6.11) | 3.5 (2.13-5.77) |
| Age | 163 | 0.97 (0.96-0.98) | 0.98 (0.97-0.99) | 0.98 (0.97-0.99) | 0.98 (0.97-1) |
| Sex: Male | 167 | 0.98 (0.75-1.29) | 1 (0.76-1.32) | 1.01 (0.73-1.39) | 0.99 (0.73-1.35) |
| Educational level: High[e] | 167 | 1.33 (0.99-1.79) | 1.34 (1-1.8) | 1.04 (0.74-1.47) | 1.02 (0.73-1.42) |
| Travels to high-risk areas: Yes[f] | 180 | 0.7 (0.51-0.95) | 0.73 (0.54-0.99) | 1.34 (0.93-1.93) | 1.27 (0.9-1.8) |
| Work with patients or human tissues: Yes[g] | 198 | 0.45 (0.31-0.63) | 0.46 (0.32-0.66) | 0.69 (0.46-1.02) | 0.66 (0.44-0.99) |
| Hospital visits as a patient: Yes | 165 | 1.07 (0.64-1.69) | 1.04 (0.65-1.66) | 1.06 (0.6-1.82) | 0.98 (0.58-1.65) |
| Hospital visits as a professional: Yes | 165 | 0.56 (0.17-1.39) | 0.57 (0.21-1.59) | 0.83 (0.23-2.33) | 0.83 (0.28-2.48) |
| Use of antibiotics: Yes | 167 | 1.08 (0.78-1.48) | 1.07 (0.77-1.48) | 1.03 (0.7-1.5) | 1.04 (0.72-1.5) |
| Farm visits: Yes | 172 | 0.37 (0.17-0.69) | 0.4 (0.2-0.77) | 0.75 (0.34-1.46) | 0.7 (0.35-1.39) |

Notes:

[d]Nearby residents live within a 300 m radius from a WWTP.

[e]Educational level according to the International Standard Classification of Education (ISCED): Low = ISCED 0-2 (Pre-primary education to Lower secondary education), High = ISCED ≥3 (Upper secondary education to Doctoral or equivalent).

[f]Travel to high risk areas for AR in the past year: Includes travels to North Africa, Sub-Saharan Africa, Asia, Central and South America, as well as the European countries Italy, Greece, Bulgaria and Slovenia.

[g]Work with patients or human tissues in the past year: Includes self-reported contact with patients at work and with human tissues (e.g. blood, urine, sputum, feces, vomit, saliva, or primary cell lines).

ESBL: Extended-Spectrum Beta-Lactamases.

AR: Antibiotic Resistance.

*Online Resource Table 4: Interaction model with an interaction term for participation group with country for the carriage of ESBL-producing E. coli in comparison to crude estimates from a traditional unweighted logistic regression model, n = 1940, AWARE Study, 2021*

| | **cOR (95% CI)[a]** | **With interaction, aOR (95% CI)[b]** |
|---|---|---|
| Group: Nearby resident [c] | 4.95 (3.63-6.73) | 0.73 (0.27-1.95) |
| Group: WWTP worker | 1.71 (1.12-2.61) | 0 (0-Inf)[d] |
| Country: Netherlands | 0.78 (0.47-1.3) | 0.79 (0.45-1.39) |
| Country: Romania | 4.85 (3.04-7.74) | 1.55 (0.79-3.05) |
| Educational level: High[e] | 1.34 (1-1.8) | 0.98 (0.7-1.37) |
| Sex: Male | 1 (0.76-1.32) | 1.03 (0.76-1.41) |
| Age | 0.98 (0.97-0.99) | 0.98 (0.97-0.99) |
| Travels to high-risk areas: Yes[f] | 0.73 (0.54-0.99) | 1.31 (0.92-1.86) |
| Work with patients or human tissues: Yes[g] | 0.46 (0.32-0.66) | 0.69 (0.46-1.04) |
| Hospital visits as a patient: Yes | 1.04 (0.65-1.66) | 0.96 (0.57-1.62) |
| Hospital visits as a professional: Yes | 0.57 (0.21-1.59) | 0.83 (0.28-2.46) |
| Use of antibiotics: Yes | 1.07 (0.77-1.48) | 1.04 (0.72-1.5) |
| Farm visits: Yes | 0.4 (0.2-0.77) | 0.72 (0.36-1.45) |
| Interaction: NL x Nearby residents | --- | [h] |
| Interaction: NL x WWTP workers | --- | 881070.99 (0-Inf)[d] |
| Interaction: RO x Nearby residents | --- | 5.49 (1.79-16.8) |
| Interaction: RO x WWTP workers | --- | 2385014.82 (0-Inf)[d] |

Notes:
[a]cOR: crude odds ratio.
[b]aOR: adjusted odds ratio.
[c]Nearby residents live within a 300 m radius from a WWTP.
[d]Not possible to estimate the OR for WWTP workers because all workers in Germany had a negative stool sample result for ESBL-producing E. coli.
[e]Educational level according to the International Standard Classification of Education (ISCED): Low = ISCED 0-2 (Pre-primary education to Lower secondary education), High = ISCED ≥3 (Upper secondary education to Doctoral or equivalent).
[f]Travel to high risk areas for AR in the past year: Includes travels to North Africa, Sub-Saharan Africa, Asia, Central and South America, as well as the European countries Italy, Greece, Bulgaria and Slovenia.
[g]Work with patients or human tissues in the past year: Includes self-reported contact with patients at work and with human tissues (e.g. blood, urine, sputum, feces, vomit, saliva, or primary cell lines).
[h]Data on Nearby residents in the Netherlands was not collected. There is, therefore, no interaction term for NL x Nearby resident.
ESBL: Extended-Spectrum Beta-Lactamases.
AR: Antibiotic Resistance.

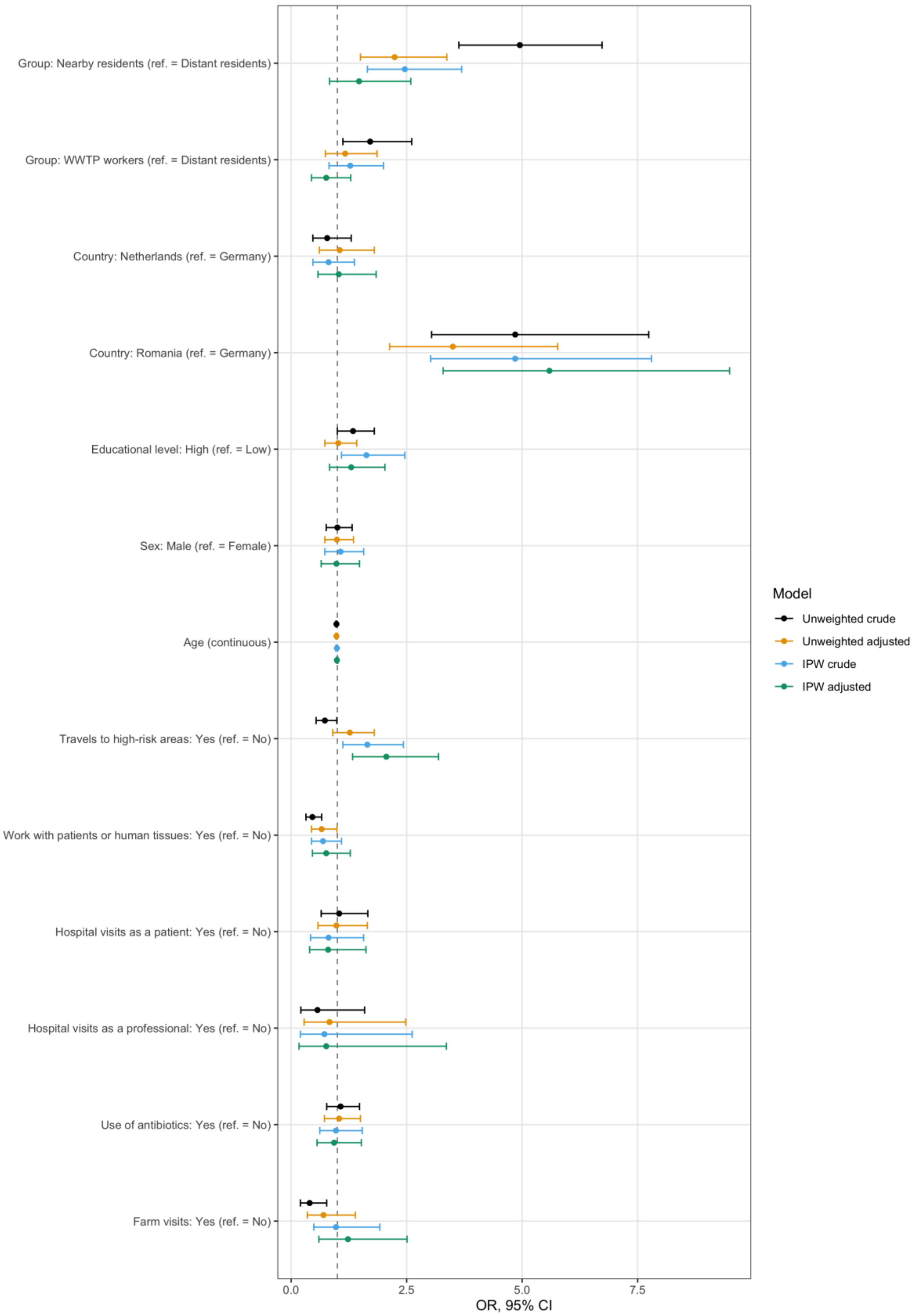


Online Resource Fig. 1: Comparison of models estimating the effect of participation group (wastewater treatment plant -WWTP-worker, nearby resident, distant resident) as a proxy for exposure routes (ingestion of droplets, hand-to-mouth contact, or inhalation of aerosols) in and around the local WWTP on the presence of ESBL-producing E. coli in stool samples (all estimates are shown), AWARE Study, 2021.

Models adjusted for age, sex, education, country, travels to high risk areas, working with human tissues, antibiotic use, farm visits, hospital visits as patient and hospital visits as a professional.

IPW: Inverse Probability Weighted model.

ref. = Reference level.
Travel to high risk areas for AR in the past year includes travels to North Africa, Sub-Saharan Africa, Asia, Central and South America, as well as the European countries Italy, Greece, Bulgaria and Slovenia.
Crude: Model with only the given variable, ignoring potential covariates.
Adjusted: Model with the given variable, including all potential covariates in the exposure-outcome relation.
Unweighted: Model without applying inverse probability weights (IPW).
Weighted: Model applying inverse probability weights (IPW).
See text for details

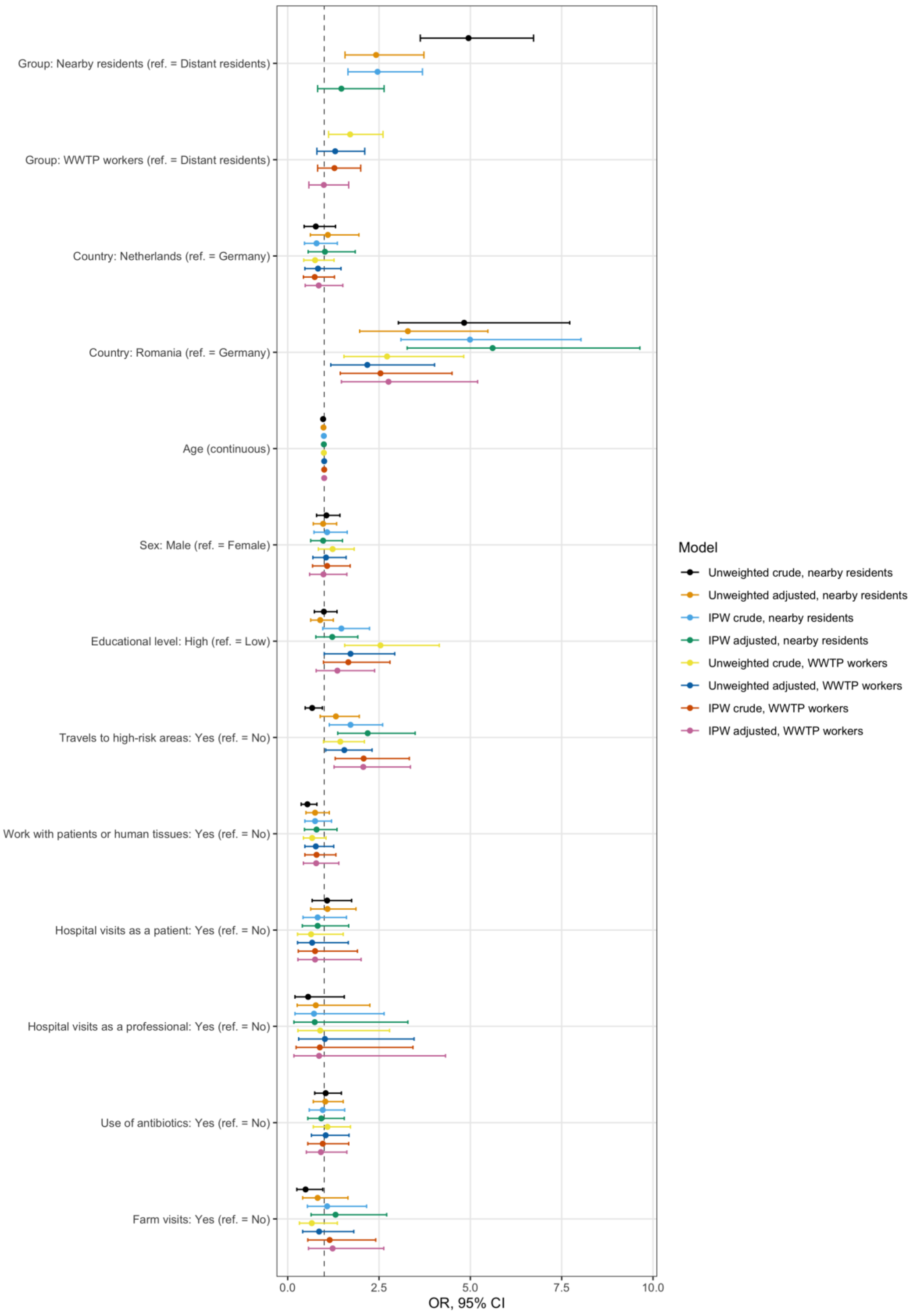


Online Resource Fig. 2: Comparison of models estimating the effect of participation group (wastewater treatment plant -WWTP- worker, nearby resident, distant resident) as a proxy for exposure routes (ingestion of droplets, hand-to-mouth contact, or inhalation of aerosols) in and around the local WWTP on the presence of ESBL-producing E. coli in stool samples, stratified by participation group, AWARE Study, 2021.
Models adjusted for age, sex, education, country, travels to high risk areas, working with human tissues, antibiotic use, farm visits, hospital visits as patient and hospital visits as a professional.
IPW: Inverse Probability Weighted model.

ref. = Reference level.
Travel to high risk areas for AR in the past year includes travels to North Africa, Sub-Saharan Africa, Asia, Central and South America, as well as the European countries Italy, Greece, Bulgaria and Slovenia.
Crude: Model with only the given variable, ignoring potential covariates.
Adjusted: Model with the given variable, including all potential covariates in the exposure-outcome relation.
Unweighted: Model without applying inverse probability weights (IPW).
Weighted: Model applying inverse probability weights (IPW).
See text for details
